# Field-Dependent Dehydration and Optimal Ionic Escape Paths for C_2_N Membranes

**DOI:** 10.1021/acs.jpcb.1c03255

**Published:** 2021-06-11

**Authors:** Miraslau L. Barabash, William A. T. Gibby, Carlo Guardiani, Dmitry G. Luchinsky, Binquan Luan, Alex Smolyanitsky, Peter V. E. McClintock

**Affiliations:** † Department of Physics, Lancaster University, Lancaster LA1 4YB, United Kingdom; ‡ Department of Mechanical and Aerospace Engineering, Sapienza University, 00184 Rome, Italy; § Ames Research Center, KBR, Inc., Moffett Field, California 94035, United States; ∥ Computational Biological Center, IBM Thomas J. Watson Research, Yorktown Heights, New York 10598, United States; ⊥ Applied Chemicals and Materials Division, National Institute of Standards and Technology, Boulder, Colorado 80305, United States

## Abstract

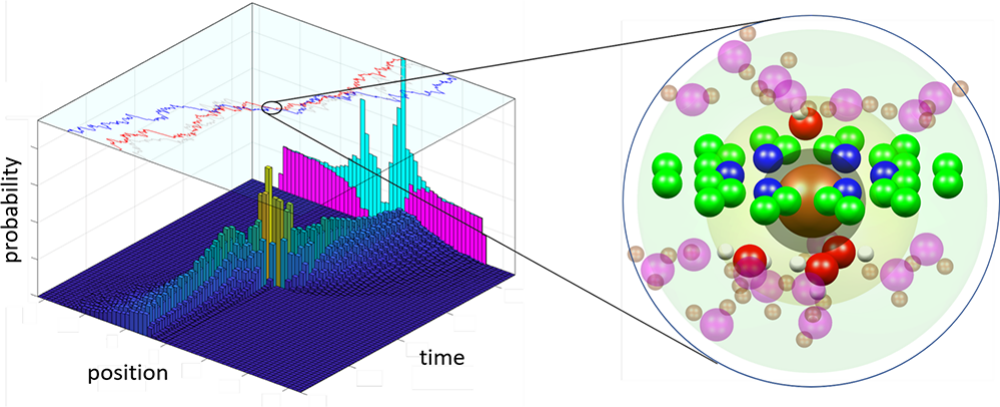

Most analytic theories describing electrostatically driven ion transport through water-filled nanopores assume that the corresponding permeation barriers are bias-independent. While this assumption may hold for sufficiently wide pores under infinitely small bias, transport through subnanometer pores under finite bias is difficult to interpret analytically. Given recent advances in subnanometer pore fabrication and the rapid progress in detailed computer simulations, it is important to identify and understand the specific field-induced phenomena arising during ion transport. Here we consider an atomistic model of electrostatically driven ion permeation through subnanoporous C_2_N membranes. We analyze probability distributions of ionic escape trajectories and show that the optimal escape path switches between two different configurations depending on the bias magnitude. We identify two distinct mechanisms contributing to field-induced changes in transport-opposing barriers: a weak one arising from field-induced ion dehydration and a strong one due to the field-induced asymmetry of the hydration shells. The simulated current–voltage characteristics are compared with the solution of the 1D Nernst–Planck model. Finally, we show that the deviation of simulated currents from analytic estimates for large fields is consistent with the field-induced barriers and the observed changes in the optimal ion escape path.

## 1 Introduction

Ionic permeation through nanopores in atomically thin membranes has attracted considerable and ever-growing attention in the past decade, for reasons that are both fundamental and practical in nature.^1,2^ There is a wide range of applications including fuel cells,^3^ water desalination,^4−8^ DNA sequencing,^9−14^ and “blue energy” harvesting.^15^ It is expected that achieving control over the permeability and selectivity of nanopores will be critically important in developing future applications.^16^ However, the problem of describing and predicting the selective conductivity of nanopores remains a formidable task.

Numerous phenomena combine to make this problem especially challenging, including the formation of water layers and electrical double layers near the membrane surface,^17^ fragmented dehydration of the ions near and inside nanopores,^18^ nontrivial variations of the local polarizability within the pore where it may differ from the bulk value by an order of magnitude,^19^ effects of polarization,^20^ and quantum mechanical interactions within the material near the pore.^21^ The complexity is further increased in the presence of a non-negligible externally applied electrostatic field, which complicates direct comparisons with analytical theories assuming near-equilibrium ensembles. Beyond nonequilibrium-related complications, external fields are expected to induce nontrivial rearrangement of hydration shells, water, and electric double layers. Because neither in realistic simulations nor in experiments can the electrostatic bias be assumed neglibible, a more complete understanding of field-induced phenomena is critical for interpretation of the experimental and simulated data.

Here we demonstrate several bias-induced phenomena affecting ionic transport through subnanometer pores by studying the molecular dynamics (MD) trajectories of ions permeating a single-layer C_2_N membrane. We focus on the effects arising due to asymmetry of the hydration shells near the pore. This asymmetry is a generic feature of the permeating ions’ local environments, as identified in earlier works,^4^ yet its effect on permeation has not been considered. Although C_2_N membranes possess the very high pore density of 1.62 × 10^18^ m^–2^, we have not observed significant effects specifically caused by the close interpore spacing. Therefore, the results presented here should be quite generally applicable to subnanometer pores in two-dimensional membranes, regardless of pore density. Moreover, the high pore density in C_2_N ensures essentially zero access resistance,^22^ thus enabling us to focus on the local barriers. The results presented below were obtained from nonpolarizable classical simulations, which are broadly applicable to nonmetallic membrane materials, including C_2_N, which are shown to be semiconducting.^23^ The results should therefore be directly relevant to nonmetallic 2D materials. At the same time, the fundamental mechanisms responsible for the bias-induced effects on the permeation barriers shown here should remain intact for electrically conductive membranes. Similarly, although we report on electrostatically driven ion transport, there remains a degree of qualitative applicability to osmotically driven systems that feature high transmembrane electric fields from local charge accumulation.

To facilitate our analysis, we use the prehistory and posthistory of ionic trajectories crossing the pores and study changes in the number and distribution of oxygen and hydrogen atoms surrounding the ions along the escape path. We show that, as the strength of the external electrostatic field is increased, the statistically significant ion escape path bifurcates and transitions to a different route. This transition correlates with the dependence of the current on the strength of the applied field. We further show that the observed behavior is closely related to the two mechanisms for bias dependence of a barrier identified here. Importantly, we demonstrate that the height of the barrier depends not only on the number of water molecules in the hydration shells, as is conventionally assumed,^24−26^ but also on the field-induced change in this number (1st identified mechanism) and on the closely related asymmetric deformation of the hydration shells (2nd mechanism). We demonstrate that the 1st mechanism is weaker than the 2nd. We note that many nanopores possess significant dehydration-associated permeation barriers, making our results widely applicable. It is therefore our hope that these findings will constitute another step toward a by-design approach to ion transport in nanofluidics.

The Article is organized as follows. In the next section we provide the details of our MD model. In section 3 we discuss the trajectories crossing the pores and their most probable paths. Unbiased dehydration barrier and the current-voltage relations are considered in section 4. The populations of the hydration shells along the escape path and the field-induced dehydration barrier are discussed in section 5. The field-induced effects on the barrier, including the asymmetry of the electric double layers and water layers near the pore, the corresponding changes in the asymmetry of the hydration shells, the resultant asymmetry of the induced transition barrier (2nd mechanism), and the current are also discussed in section 5. Finally, we summarize and provide conclusions for this work in section 6.

## 2 Model

**Figure 1:**
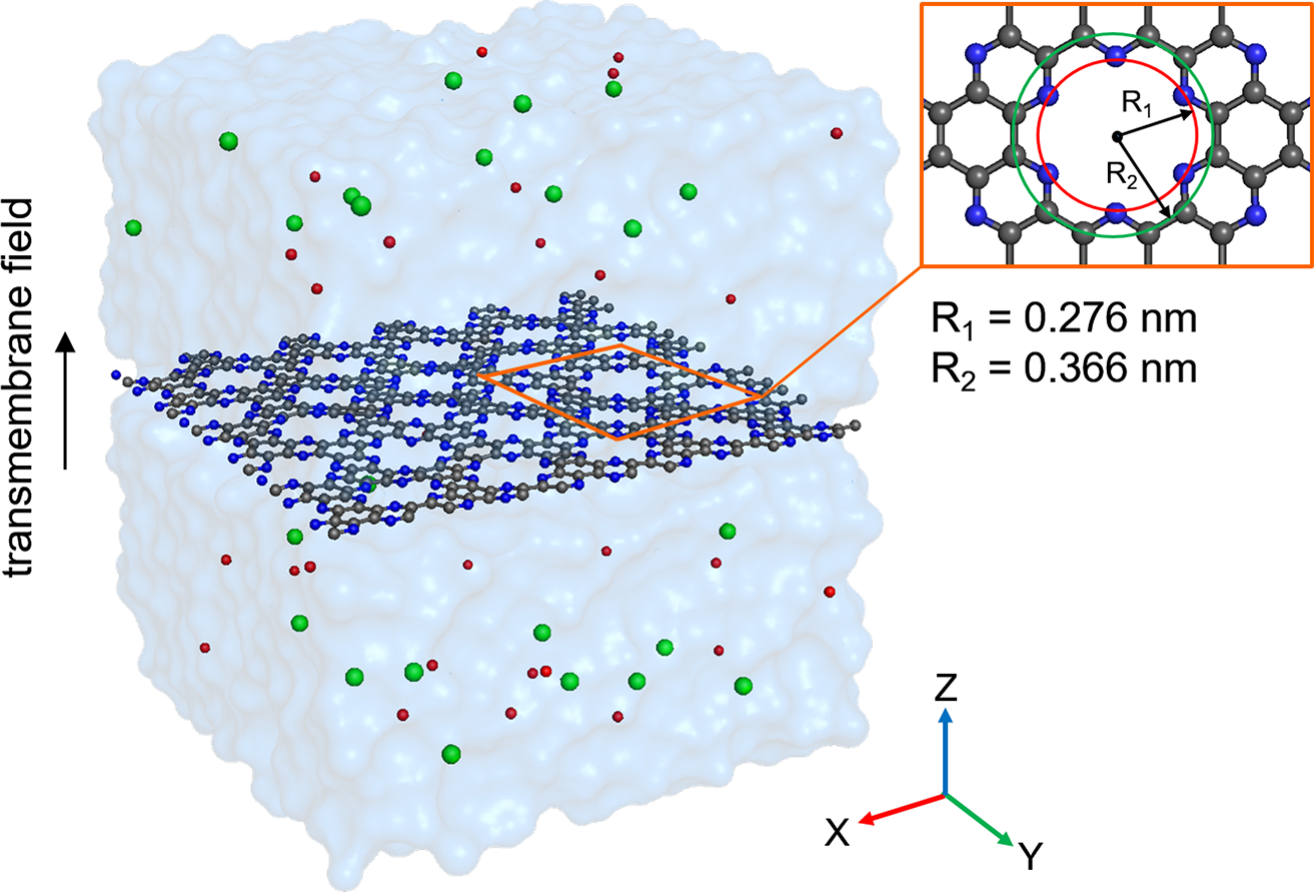
Model of a C_2_N membrane immersed in aqueous KCl solution. The nitrogen and carbon atoms in the C_2_N lattice are shaded blue and dark gray, respectively. Water is shown as a blue transparent surface; potassium and chloride ions are shown as red and green balls, respectively.

We consider a single layer of C_2_N^27−30^ immersed in 0.5 M aqueous potassium chloride, as sketched in Figure 1. The choice of potassium chloride is for consistency with a wide range of earlier experimental work, as well as due to its natural abundance and biophysical relevance. Note that, although this work focuses on the effect of field-induced asymmetry of the hydration shells during cation permeation through cation-selective pores, the fundamental mechanisms responsible for the permeation barriers are expected to be the same for anions permeating anion-selective pores, thus suggesting qualitative applicability of our results regardless of ion charge.

The partial atomic charges in the C_2_N lattice, obtained using density functional theory calculations set up according to earlier work,^31,32^ are −0.31*e* for nitrogen and +0.155*e* for carbon atoms. The Lennard-Jones parameters were set according to the OPLS-AA force field^33^ (σ_NN_ = 0.325 nm, ϵ_NN_ = 0.71128 kJ/mol; σ_CC_ = 0.355 nm, ϵ_CC_ = 0.29288 kJ/mol). Aside from the harmonically restrained atoms at its perimeter, the C_2_N membrane was simulated as fully flexible. The corresponding bonded parameters for C_2_N and the rest of the simulation components were set up according to the standard OPLS-AA^33^ framework. To study the permeation trajectories of potassium ions, a static electric field was applied in the direction normal to the plane of the membrane with a strength varying between 0 and 300 mV/nm. The rectangular simulation box size was 4.38 × 4.22 × 5.0 nm, where the membrane was located in the *xy* plane at *z* = 2.5 nm and featured 30 periodically positioned nanopores with an effective spacing of 0.551 nm (see Figure 1). Typical counts of C_2_N atoms, water molecules, and dissociated salt ions in the system were 540, 2750, and 60 (30 on each side), respectively. Prior to production simulations, all systems were subject to relaxation in the semiisotropic NPT ensemble (box size constant in the *XY* plane, barostat-controlled *Z*). The production simulations were performed in the NVT ensemble. All simulations were performed with periodic boundaries applied in *XYZ* and were carried out using GROMACS ver. 2018.1.^34−36^ For production simulations, a time step of 2 fs was used, and trajectories were tracked for at least 300 ns at each value of the applied electric field. The TIP4P water model was used,^37,38^ yielding a bulk dielectric constant of ε ≈ 53. All ion trajectories crossing the nanopores were collected. The positions of oxygen and hydrogen atoms within two hydration shells of the ion were recorded at given distances from the pore corresponding to the escape process.

Our statistical analysis of these trajectories and the corresponding distributions of ions and water molecules in the system as a function of the applied electric field will now be presented and discussed.

## 3 Trajectories and Prehistory Probability Distribution

### 3.1 Trajectories

**Figure 2:**
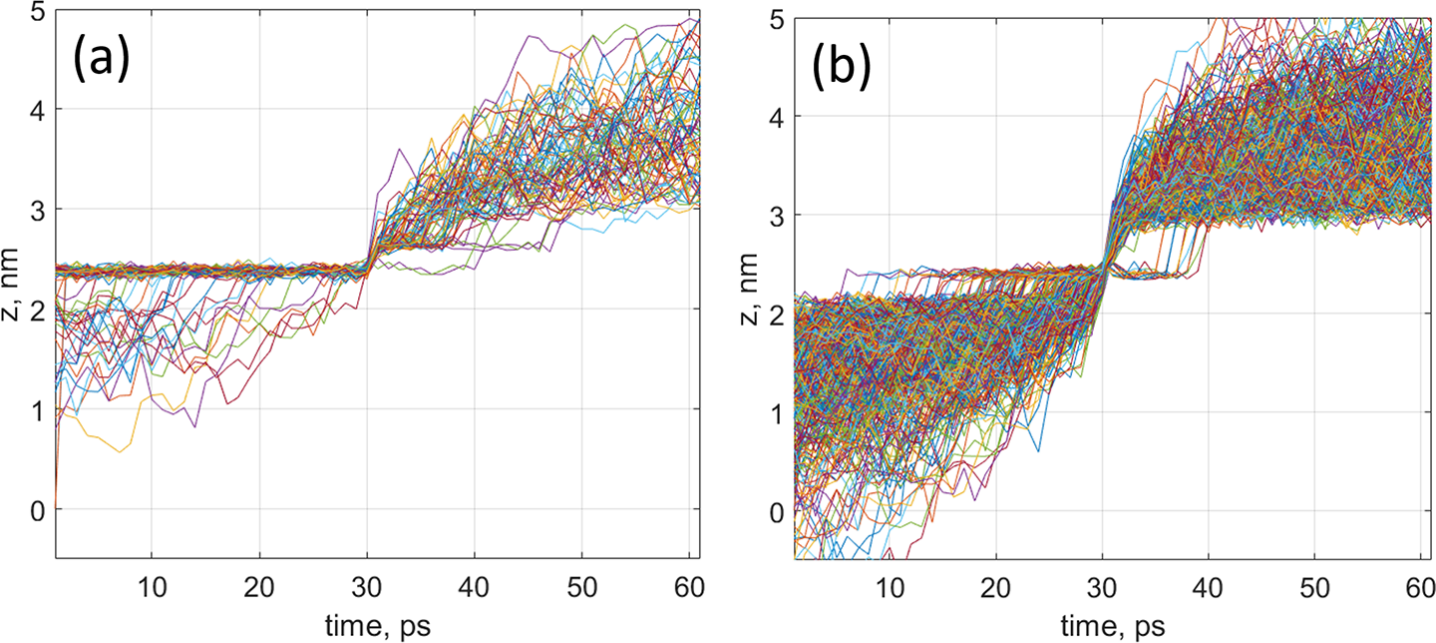
Molecular dynamics trajectories crossing the pore for applied fields of (a) 50 mV/nm and (b) 200 mV/nm.

Typical examples of trajectories obtained in MD simulations for potassium ions crossing the pores are shown in Figure 2 for electrostatic field values of 50 mV/nm (a) and 200 mV/nm (b). It can be seen from the figure that the ions dwell at a well-defined location at *z* = 2.4 nm (0.1 nm below the membrane plane) prior to their escape through the pore to the other side of the membrane. After the escape, ions appear to dwell at *z* = 2.6 nm (0.1 nm above the membrane plane). As the applied field is increased, the pre- and postescape locations of the escaping ions symmetrically shift away from the membrane to ∼2.0 and ∼3.0 nm, respectively. Unsurprisingly, the postescape location is reached rapidly. These changes in trajectory with increasing field depend on many factors, including the dehydration of the ions and the structure of the electric double layers, and these are considered in more detail below.

### 3.2 Prehistory

**Figure 3:**
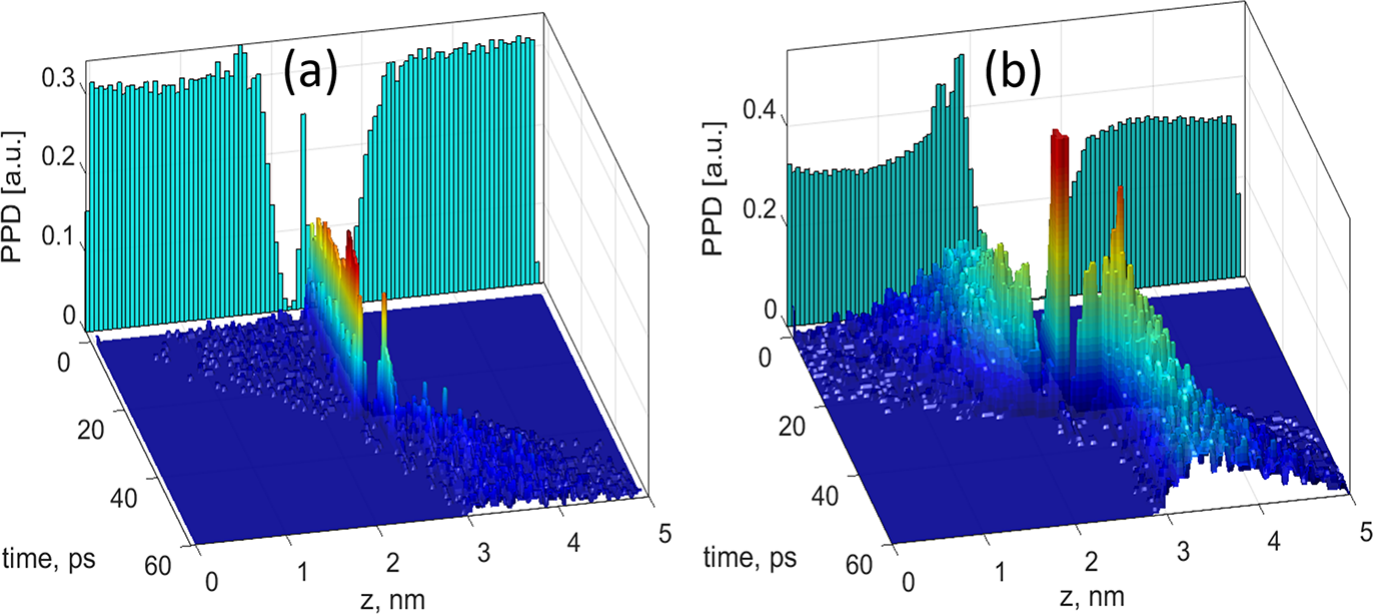
Prehistory probability distribution obtained using molecular dynamics for applied fields of (a) 0.05 V/nm and (b) 0.2 V/nm.

Initial insight into the ionic permeation process can be obtained from an examination of escape trajectories. The statistically significant structure of these trajectories can be obtained from the prehistory probability distribution (PPD).^39−41^ The underlying concept is that the probability of observing an ion escaping near the boundary *x*_f_ of the attractors of two metastable states is small, due to the relatively high transition barrier separating them. In this system the boundary separates the pre-escape and postescape bulk solutions. Here, the pre-escape bulk solution corresponds to the bulk solution below the membrane shown in Figure 1. Note that cations escape in the direction of the field, i.e., to the postescape region above the membrane. In Figures 2 and 3, the state transition from pre- to postescape occurs within ∼30 ps. The time intervals between successive escape events are relatively large, and they are expected to exceed the system’s characteristic relaxation time for reaching quasi-equilibrium. Thus, escape events are mutually uncorrelated, so that the PPD can be expressed via the probability density functional  of the trajectory *x*(*t*) of the system.^42^
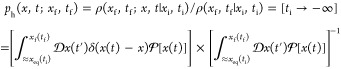
1where ρ(*x*_f_, *t*_f_; *x*, *t*|*x*_i_, *t*_i_) is the conditional probability density for a system placed initially at *x*_i_ to pass through the states *x* at the instants *t* and arrive to the state *x*_f_ at the final time *t*_f_, while ρ(*x*_f_, *t*_f_ |*x*_i_, *t*_i_) is the two-time transition probability. To eliminate dependence of the PPD on the initial state, the limit being taken is that in which the initial instant *t*_i_ goes to −∞. The importance of the initial state (*x*_i_, *t*_i_) in the definition of the prehistory probability distribution can be understood if we recall that the particle can arrive at the barrier (*x*_f_, *t*_f_) from different basins of attraction. For example, in our system there are two such basins—the two bulk solutions. Accordingly, the limit (*t*_i_ → −∞) has to be taken with care to ensure that the particle remains within the same basin of attraction at all times during the analysis.

Empirically the PPD can be found by collecting all the trajectories that move the system to the state *x*_f_ from a given basin of attraction, setting the final time for all trajectories to a fixed value *t*_f_ and building the distribution of all such trajectories. This concept has been shown to be useful for analysis of the dynamics of comparatively rare fluctuational escape events in systems that are locally far from thermal equilibrium.^40,43^

To apply this concept to the ions’ escape through C_2_N, we note that all escape trajectories have a clearly resolved time marker corresponding to the instant an ion is located in the plane of the membrane. Also, as noted earlier, escape events are well-separated in time, i.e., while the transition of one ion takes ∼100 ps, the time interval between transitions is considerably longer, on the order of nanoseconds. It then becomes possible to collect the escape trajectories of K^+^ ions and superimpose them using the escape time marker, thus separating the pre- and postescape portions of all trajectories. Finally, the 2D histograms of the superimposed trajectories are built to reveal the expected paths for ions to approach the membrane (prehistory) and to leave it on the other side of the pore (posthistory). The resulting prehistory probability distributions are shown in Figure 3 for applied fields of 50 and 200 mV/nm. We can see that there exists a most probable escape path (MPEP) for ions approaching the pore in each case. Interestingly, the location of the MPEP shifts as the applied field is increased, in accordance with the results shown in Figure 2. It also can be observed that the location of the ridge of the MPEP coincides with the peaks of the K^+^ ion distributions shown on the vertical back planes of Figure 3 by blue bars. Finally, we note that an ion is ejected quickly from the pore into the other bulk, as soon as it has crossed the membrane plane.

The dynamics of ionic permeation is strongly affected by the potential of the free energy landscape^44^ and by the applied electric field, as will be discussed below.

## 4 Field-Independent Hydration Shells

Here we consider the connection between ionic permeation and the potential of the mean force (PMF). Following convention,^45−47^ we assume that the PMF can be decomposed into components related to the dehydration, Lennard-Jones, and electrostatic interaction energies. We also assume that the effective total potential is the sum of the external electric potential, the single-ion PMF, and other terms to take account of long-range electrostatic interactions in an inhomogeneous dielectric environment.^48,49^ Within this approach we consider dehydration to be a function of the average number of water molecules in the hydration shells of the permeating ion.^24^ We will summarize the limitations of the conventional approach at the end of this section and will present the results of an extended analysis in section 5.

### 4.1 Potential of the Mean Force

**Figure 4:**
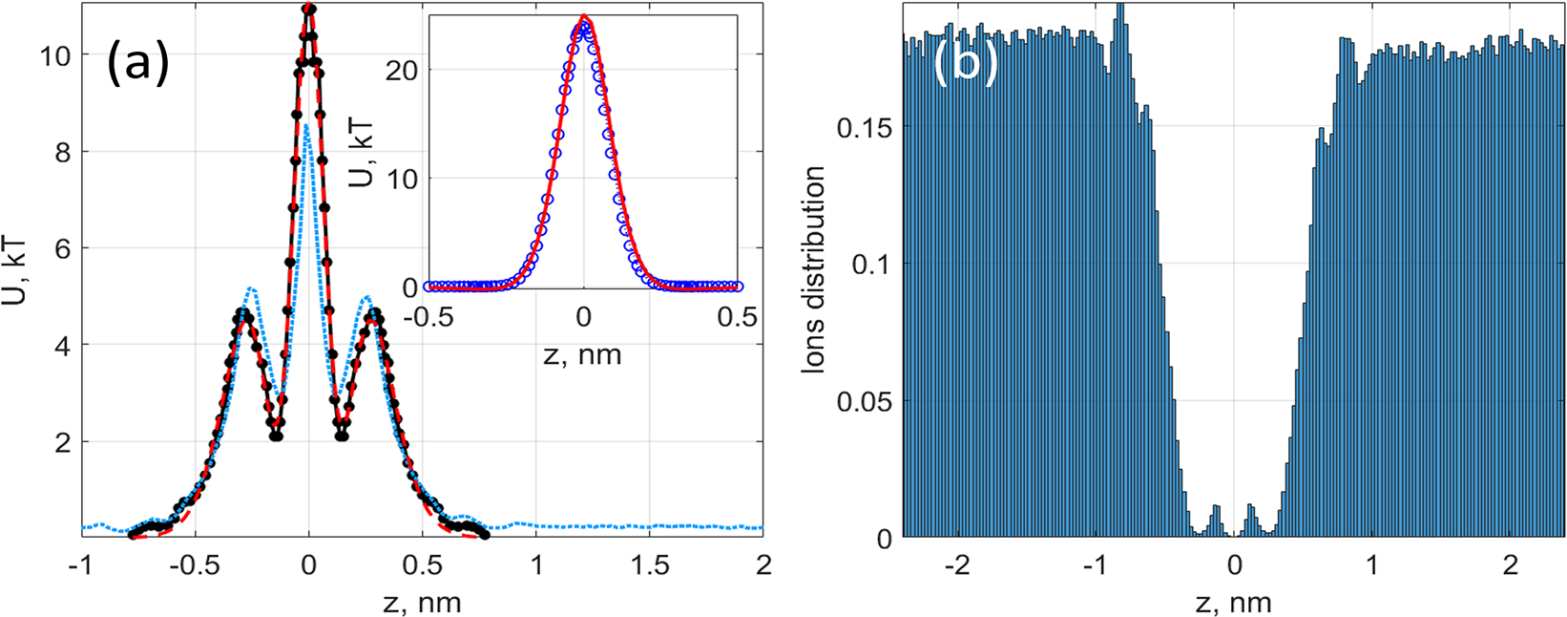
(a) Potential of the mean force obtained in MD (black solid line with black-filled circles) simulated at zero applied field using WHAM, compared to −log(ρ(*z*)) (blue dotted line) and an analytic fit of the PMF with three Gaussians (red dashed line). The inset compares the Gaussian fit (blue open circles) with exact MD calculations (red solid line) of the Lennard-Jones potential. (b) Distribution of potassium ions ρ(*z*). For clarity, the *z* coordinate has been shifted by 2.5 nm to bring the pore location to *z* = 0 nm.

As mentioned earlier, the dynamics of the permeation process is strongly affected by the free energy landscape. For zero and small applied fields, the Gibbs free energy as a function of the effective reaction coordinate can be approximated by the PMF shown in Figure 4a. The PMFs in this work were calculated by combining umbrella sampling simulations with the weighted histogram analysis method (WHAM).^50^ The sampling was obtained from disk-shaped bins located at a series of *Z* distances from the membrane, with each bin parallel to it in the *XY* plane. The (*X*, *Y*) location of ions restrained in the *Z* direction within each bin was sampled stochastically to obtain the corresponding histograms. For the PMFs shown in this work, the effective reaction coordinate was the ions’ *Z* separation from the membrane plane.

A comparison of the PMF with the trajectories (Figure 2) and prehistory distributions (Figure 3) shows that the ions’ dwelling locations correspond to the two local minima of the PMF. The applied field is forcing ions in the left-most minimum to escape through the pore. However, the high central potential barrier (∼10*kT*) keeps the ions from escaping from this local minimum for a relatively long time time, as compared to the time required to populate it. For zero applied field, the locations of these minima can be found using the Boltzmann distribution as^51^
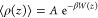
where β = 1/*kT* and *W*(*z*) is the PMF; see Figure 4a. It is generally expected that the PMF should correspond closely to the natural logarithm of the distribution for K^+^ ions in the system as a function of distance from the membrane −log(⟨ρ(*z*)⟩). Indeed, a close correlation between the PMF and −log(ρ(*z*)) can be seen in Figure 4a.

The distribution of K^+^ ions, ρ(*z*), in the absence of a biasing field was obtained using 800-ns-long simulations by dividing the simulation box into 202 bins in the *z* direction. The deviation of −log(ρ(*z*)) from the PMF at the central peak location is attributed mainly to the well-known problem of poor sampling of regions of high potential energy, with only a few counts per bin at the barrier location. As mentioned earlier, it is customary to present the PMF as a sum of components arising from different physical interactions. In particular, here the three peaks and two minima of the PMF are attributable to the interplay of three main physical mechanisms: (i) the dehydration of the ion while passing through the pore; (ii) the Coulomb interaction of the ion with the charged atoms of the membrane; and (iii) the Lennard-Jones (LJ) interaction of the ion with the rim atoms. We note that the LJ contribution does not depend on the electrostatic interaction or on dehydration. Within the conventional approach,^24,52^ the ion’s dehydration is a function only of the pore geometry (see next subsection). The simplest assumption, that the three components are mutually independent, is therefore reasonably justified, and the overall PMF is expected to be their sum.

To illustrate this point, we fit the PMF curve using three Gaussian curves representing the three distinct interaction mechanisms mentioned earlier, as shown in Figure 4a. All of our Gaussians are centered at the *z* position corresponding to the membrane location (*z* = 0).
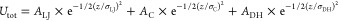
2We use the following values of the amplitude *A* and standard deviation σ: *A*_LJ_ = 23.9*kT*, σ_LJ_ = 0.078 nm; *A*_C_ = −36.4*kT*, σ_C_ = 0.13 nm; and *A*_DH_ = 23.51*kT*, σ_DH_ = 0.19 nm for the Lennard-Jones (LJ), Coulomb (C), and dehydration (DH) interactions, respectively.

It is clear from the fitting results that the contributions of these different mechanisms have comparable magnitudes (note, however, the negative sign of the Coulomb contribution) and similar values of standard deviation. The repulsive Lennard-Jones component is spatially narrow, as expected, while the two minima at the sides result from the sum of the dehydration and Coulomb contributions. We emphasize that the overall PMF can be nonuniquely constructed from a variety of Gaussian curves, and thus the considerations mentioned earlier are provided mainly for qualitative illustration.

### 4.2 Lennard-Jones Interaction

To further calibrate this approach for semiquantitative analysis, we compare the LJ component provided by our Gaussian fit (the 1st term in eq 2) with the results of explicit calculations based on the parameters (ϵ_*ij*_ and σ_*ij*_; see eq 3) of the LJ interactions taken from the corresponding OPLS-AA force field entries. The LJ interaction energy is calculated as a function of the ion’s distance *z*_*i*_ from the pore using the a priori known locations of the carbon and nitrogen atoms
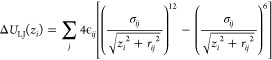
3Here *r*_*ij*_ is the distance from the *i*th ion to the *j*th carbon or nitrogen atom in the membrane plane. The LJ contribution was selected for calibration because it does not depend on the Coulomb or dehydration components, which themselves are nontrivially interrelated in general. The fitted LJ contributions are compared with the exact MD results in the inset of Figure 4a. Details of the fitting will be given elsewhere. Finally, the small oscillations observed in the tails of the distribution between 0.5 and 1 nm are attributable to the double layers of the K^+^ ion distributions that are clearly seen in Figure 4b.

### 4.3 Ion Dehydration in the Pore

**Figure 5:**
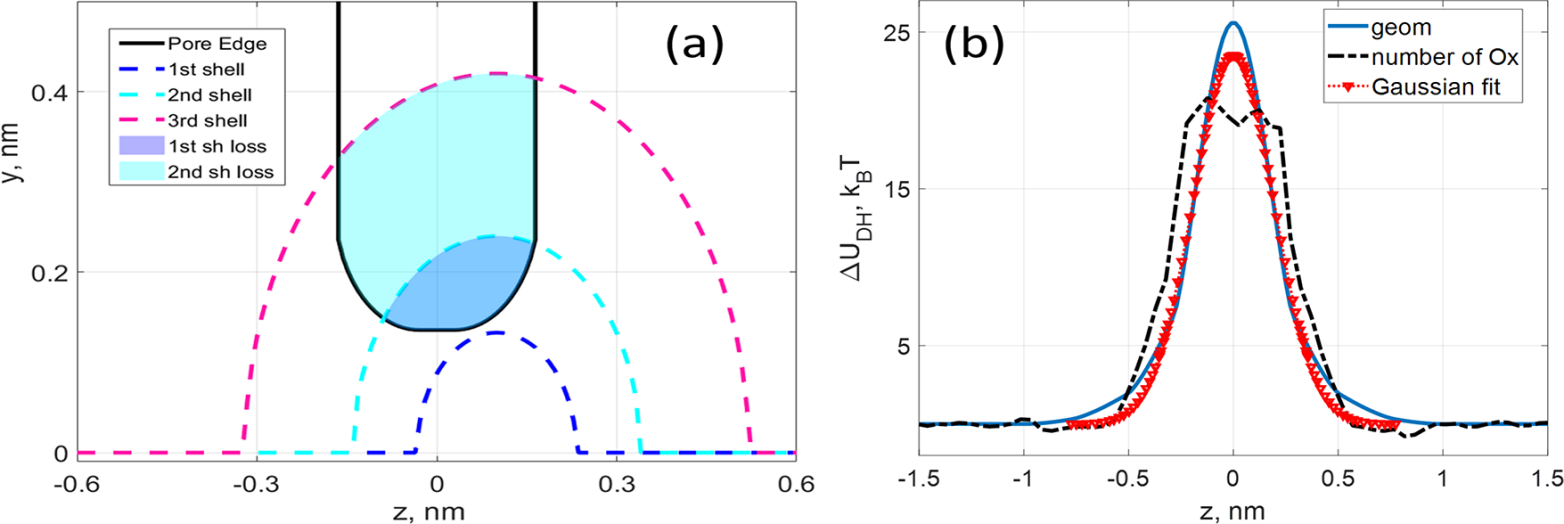
(a) Dehydration of a K^+^ ion during its passage through the pore. The pore profile is shown by a black line, the inner radius of the first shell is shown by a blue dashed line, the inner radius of the second shell (i.e., the outer radius of the first shell) is shown by a cyan dashed line, and the outer radius of the second shell (i.e., the inner radius of the third shell) is shown by a red dashed line. The volumes lost from the first and second shells are shaded in blue and cyan. (b) Dehydration potential barriers obtained by calculating (i) dehydration volumes (blue solid line); (ii) analytic fit (red triangles) using eq 2; and (iii) MD calculations of the number of oxygen molecules (Ox) in the hydration shells (black dashed line).

A key phenomenon affecting ion transport through sufficiently narrow nanopores is transient dehydration, which occurs when ions must temporarily shed a significant portion of their hydration shells in order to pass through the pore. As a result, the local ion–solvent interactions weaken temporarily, causing a permeation-opposing energy barrier. In Figure 5a, the boundaries of the first three hydration shells of a K^+^ ion approaching a C_2_N pore are shown as dashed lines, while the pore boundary is shown as a solid black line. The cross sections of the hydration shells being cut off by the pore region are color-shaded.

Rough estimates of the resultant dehydration barrier can be obtained analytically as described earlier.^5,24^ For instance, the energy contributed by each hydration shell can be estimated as^24^
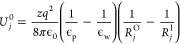
4where *R*_*j*_^O^ and *R*_*j*_^I^ are the outer and inner radii of the shell, respectively; ϵ_0_, ϵ_p_ = 3, and ϵ_w_ = 53 (for the TIP4P water model) are the permittivities of vacuum, membrane, and water, respectively. The values of the *R*_*j*_^O,I^ were taken from ref (24) to be 0.19, 0.38, 0.62, and 0.84 nm for the first three shells. The dehydration barrier is then calculated as the sum
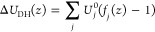
5where *f*_*j*_(*z*) is the remaining geometrical fraction of the shell available to water molecules *j* at distance *z* from the membrane along the pore axis. The estimated dehydration barrier as a function of *z* is shown in Figure 5b as a solid blue curve. For comparison, we also show the results of the analytical fit (the last term in eq 2) with red triangles. The black dashed lines denote the dehydration barrier estimations based on the average number of water molecules in the ion’s hydration shell as it permeates through the pore. For further details of these calculations, see section 5.2.

**Figure 6:**
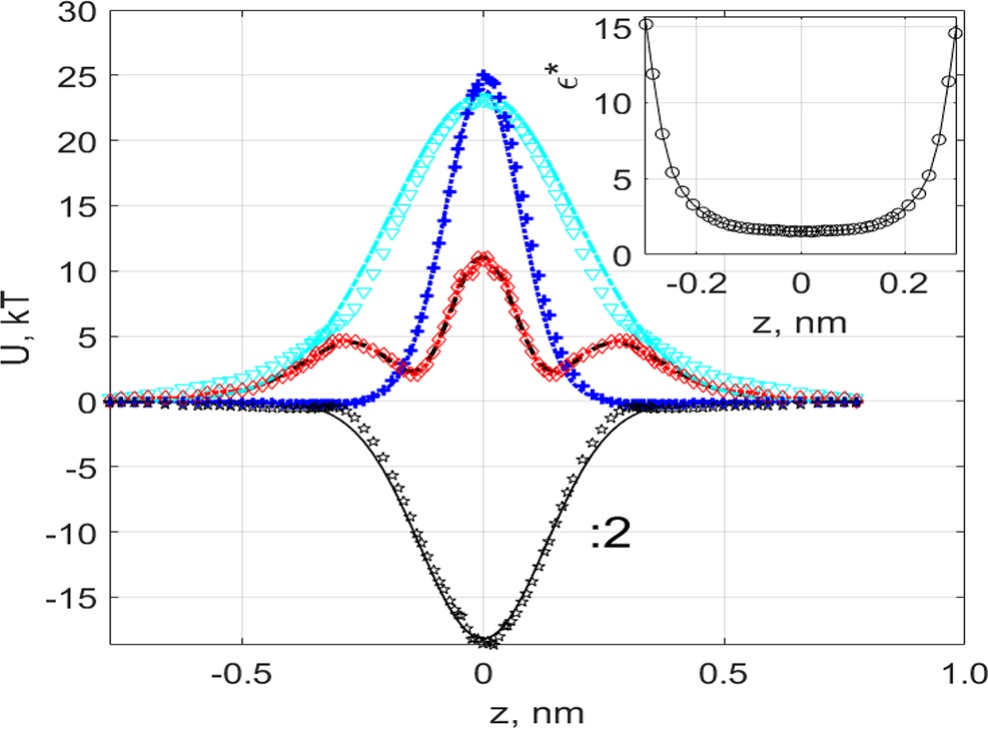
Results of triple-Gaussian fit of the PMF using eq 2. The overall PMF fit is shown by a black dashed line in comparison with the MD results depicted as red diamonds. The Gaussians are shown by lines for the following contributions: (i) dehydration, cyan dashed–dotted line; (ii) LJ, blue dotted line; and (iii) Coulomb, black solid line. The corresponding theoretical approximations are shown by symbols: (i) Δ*U*_DH_ (eq 5), cyan triangles; (ii) Δ*U*_LJ_ (eq 3), blue plus signs; and (iii) contribution due to Coulomb interactions (*U*_Coul_ = *U*_tot_ – Δ*U*_LJ_ – Δ*U*_DH_), black pentagons. The Coulomb contribution has been halved to balance all the contributions in the figure. The inset shows an estimate of the effective dielectric permittivity of the channel as ϵ* = *U*_Coul,vac_/*U*_Coul_.

It can be seen from Figure 5b and the inset of Figure 4a that the Gaussian approximations fit quite well both the Lennard-Jones Δ*U*_LJ_ and the dehydration Δ*U*_DH_ contributions to the total PMF. As mentioned earlier, within the conventional approach, the latter contributions are exact and independent, so that the decomposition of the PMF into three additive components is well-justified. The final fit is shown in Figure 6. There is good agreement between the Gaussian approximation and the corresponding theoretical results for each component and for the overall PMF.

We emphasize that, despite the good agreement between the simulated results and the additive Gaussian fits, the data presented should be considered only as rough estimates for the dehydration and Coulomb components. Specifically, the dielectric permittivity in eq 4) is assumed to be constant, and the two contributions are assumed to be independent. Within the pore, neither of these assumptions is accurate.^53^ In fact, this can be shown by estimating the effective dielectric permittivity within the pore as ϵ* = *U*_Coul,vac_/*U*_Coul_. Here *U*_Coul,vac_ is the exact potential of the Coulomb interaction of the ion with the pore in vacuum, and *U*_Coul_ = *U*_tot_ – Δ*U*_LJ_ – Δ*U*_DH_ is the same potential in the solution. The resultant value of ϵ*, shown in the inset of Figure 6, is qualitatively consistent with earlier work.^53^ The effective permittivity averaged between ±0.3 nm of the effective pore height is ∼3, which is the value used for ϵ_p_ in eq 4. Estimation of the PMF components can be further refined iteratively. For example, an iteration of Δ*U*_LJ_ can be obtained by substituting ϵ_p_ with the spatially distributed ϵ* (inset of Figure 6). This substitution will modify Δ*U*_LJ_, which will in turn modify Δ*U*_DH_. This iterative procedure can be continued in a self-consistent manner until convergence of the corresponding PMF components is obtained. It should thus be clear that the Gaussian-based approximations presented here provide only a zeroth-order estimation. In the next section, we will discuss the coupling of the two contributions with each other and with the external field.

### 4.4 Current–Voltage (*I*–*V*) Relationships

**Figure 7:**
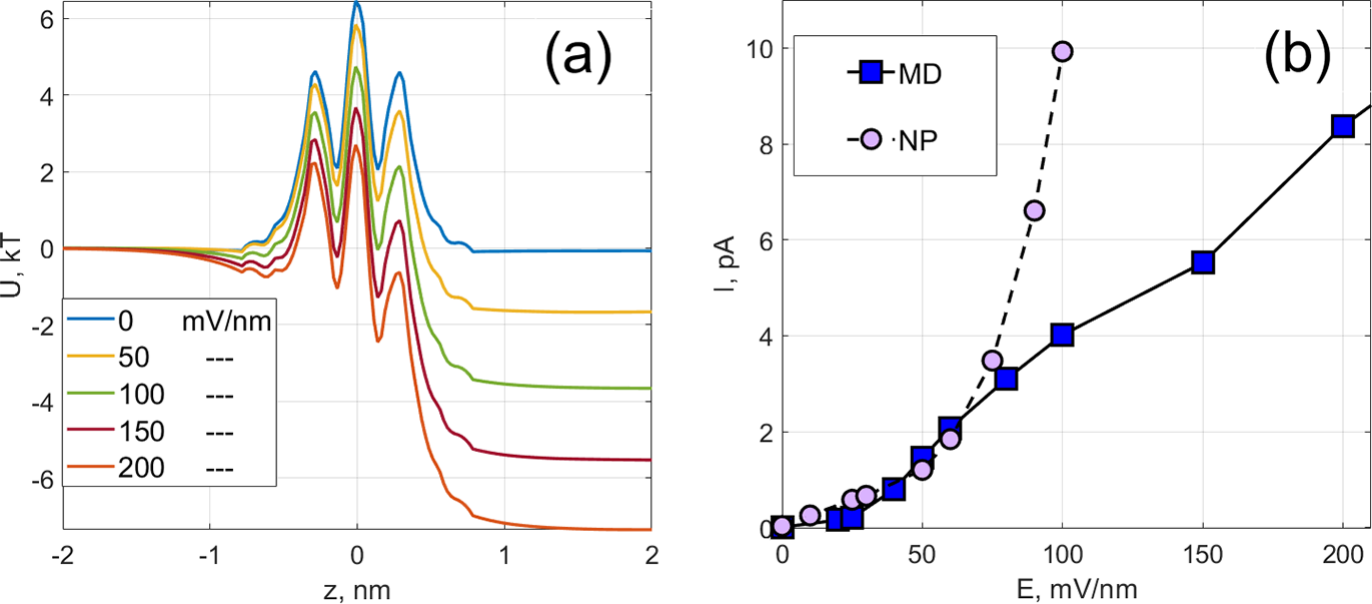
(a) Ion’s electrochemical potential for various magnitudes of the applied field shown in the legend in mV/nm. (b) Comparison between currents obtained from MD simulations (blue squares) and the solution of the 1D NP equation (eq 8) (pink shaded circles).

In MD simulations, the ionic currents through the pores were obtained using two independent methods. In the first method, we measured the displacement of all ions moving along the *z* direction during a given time interval.^54^ In the second, we tracked all ion transitions through the pore.^5,25^ These are shown as trajectories in Figure 2. The resultant current–voltage dependence is shown in Figure 7b. The two methods yield data within the uncertainties associated with thermal fluctuations, and the corresponding results are combined in the figure.

Knock-on events were not detected between ions passing through pores and their mobile neighbors. The knock-on process requires that an ion dwells inside a pore for a period longer than that required to experience at least one collision with another cation. However, the corresponding collisional frequency is reduced due to the local electrostatic repulsion between cations, similar to the case of ion-trapping crown ether-like pores in graphene.^25^ Importantly, we did not detect any instances of an ion being within 1 nm of another ion that was permeating the pore. Transition events appeared to be independent and separated in time by at least 0.5 ns for large applied fields (≥200 mV/nm) and by ∼5 ns for applied fields on the order of 50 mV/nm. Because we observed no significant ion–ion coupling during the permeation events, the use of coupling-free models to estimate the current–voltage dependence appears warranted here.

One such model is based on the 1D Nernst–Planck (NP) theory,^46,55^ according to which the current of a single conducting species (e.g., K^+^) through the pore is determined by the concentration gradient (*ñ*(*z̃*)) and the sum of the electrostatic potential (ϕ) and the PMF (*W*^PMF^) across the pore in dimensional units:^46^
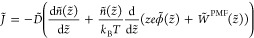
6Here, *ñ* is the number concentration of K^+^ ions normalized by the bulk concentration, ϕ̃ = ϕ*U*_*T*_ and *W̃*^PMF^ = *W*^PMF^*U*_*T*_, where *U*_*T*_ = *k*_B_*T*/*e* and *z̃* = *zd*, where *d* is the distance between left and right boundaries.

We note that, when an external field is applied, the resulting effective total potential within the conventional approach is assumed to be the sum of the external electric potential, single-ion PMF, and other terms that account for the long-range electrostatic interactions in the inhomogeneous dielectric environment.^48,49^ Such an approximation can be used for zeroth-order estimation of the current–voltage relations in our system, as we now discuss. To further simplify the estimations for consistency with this zeroth-order approximation, we assume that, within the channel, the dielectric environment is homogeneous and the corresponding effective dielectric permittivity is ϵ* ≈ 3, as mentioned earlier in Figure 6 caption and the associated discussion. Approximating the potential profile as a sum of the equilibrium PMF obtained from MD simulations (Figure 4a) and the corresponding voltage drop due to the external field, we get the following,
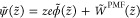
7where the voltage drop was calculated using the built-in utilities of the GROMACS package. The resulting electrochemical potential is shown in Figure 7a.

By integrating the NP equation along *z*, one arrives at^46^
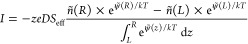
8

Ionic currents estimated using eq 8 are compared with the results of MD simulations in Figure 7b. We can see that the simplified picture of the electrochemical potential (eq 8) can capture a number of important features of the problem. In particular, the central barrier decreases while the new potential minimum appears at location −0.75 nm. As a result, the NP model allows us to reproduce the initial activation-type increase of the current–voltage curve, as illustrated in Figure 7b. However, the simple NP model does not capture the deviation from the activation regime of the current–voltage curve observed for an applied field >70 mV/nm. In addition, it does not explain the transition of the MPEP discussed in subsection 3.2 to the new location, because the old location at approximately −0.135 nm remains the deepest minimum on the left side of the membrane. This demonstrates the fundamental inaccuracy of NP-based calculations using an artificially perturbed equilibrium PMF (Figure 7a). Therefore, understanding the physical origin of the nonperturbative nature of a realistic PMF under an external field is critically important. We will now show that further insight into phenomena affecting the energetics and transport can be gained by analyzing the field-dependent dehydration and asymmetry of the hydration shells around the ion.

## 5 Asymmetry of Hydration Shells and Field-Induced Barrier

We now consider two phenomena that accompany ionic transitions through the pore under an external field: the field-induced changes of the average number of water molecules surrounding the permeating ion and the field-induced asymmetry of the corresponding hydration shells, including those away from the immediate vicinity of the pores. Both phenomena will be shown to induce an increase in the transition barrier. We discuss this in detail and estimate the corresponding field-induced transition barriers.

We perform the analysis in two steps. First, we present the results showing the field-induced asymmetry of the hydration shells. Next, we calculate the forces acting upon ions within the asymmetric shells and estimate the resultant contribution to the transition barrier. Finally, we show that the observed current–voltage relationship and the changes in the corresponding MPEPs are consistent with these field-induced effects.

### 5.1 Ions/Water Layering and Symmetry

The effect of external bias on an ion’s passage through a subnanoscale pore is multifaceted. In addition to the first-order influence of the field on the ions’ dynamics discussed earlier, the electrical bias also modifies water layering, electric double layers, and the structure of the hydration shells in and near the pore. These effects are in addition to the field-induced concentration gradients and asymmetric charge accumulation at the two membrane surfaces.

**Figure 8:**
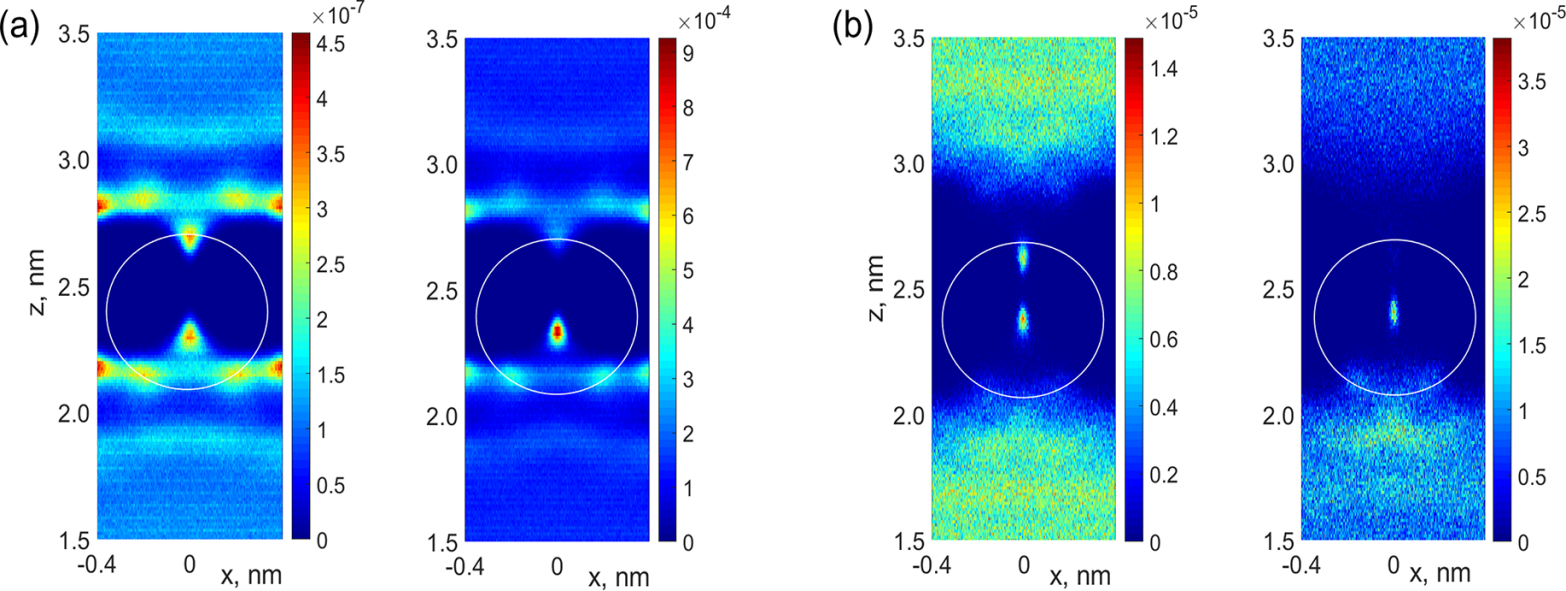
(a) Calculated distribution of water oxygens in the *xz* plane for an unbiased system (left) and with an externally applied field of 200 mV/nm (right). (b) Potassium ion distribution near the pore for two different applied electrostatic fields: 0 mV/nm (left) and 200 mV/nm (right). The white circles indicate the location of the first hydration shell centered around the peak in the island of ions distributed below the pore.

Here we consider the field-dependent layered structures that form near the membrane surfaces and affect ion transport. Predicting these structures is a nontrivial problem, especially in the presence of charged or dipolar pores. An example of the distribution of the water oxygens near the pore is shown in Figure 8a. Water layering is evident, consistent with earlier findings.^4,56^ We also observe that these layers are fragmented near the pores, with islands of increased oxygen density likely near the positively charged carbon atoms in C_2_N. When an external electrostatic field is applied, the peak of the oxygen distribution in the island directly below the pore increases significantly (note the different intensity scales in the left and right panes of Figure 8a), while the similar peak on the opposite site decreases. These changes are strongly correlated with the distribution of K^+^ ions near the pore, as can be seen by comparing parts a and b of Figure 8.

For K^+^ ions, similar islands of symmetric high concentration above and below the pore can be observed in both distributions for the unbiased system. The peaks above the pores almost completely disappear under a high applied field. It further can be noticed that the positions of the three peaks in the oxygen distribution coincide with the location of the 1st hydration shell of ions confined at the island below the pore. The symmetry of this distribution is broken once again under external bias. It is expected (and confirmed by MD simulations, as discussed in the next section) that the broken symmetry of the water and ion distributions will also result in a strong asymmetry of the hydration shells with respect to the membrane plane.

### 5.2 Field-Induced Dehydration

**Figure 9:**
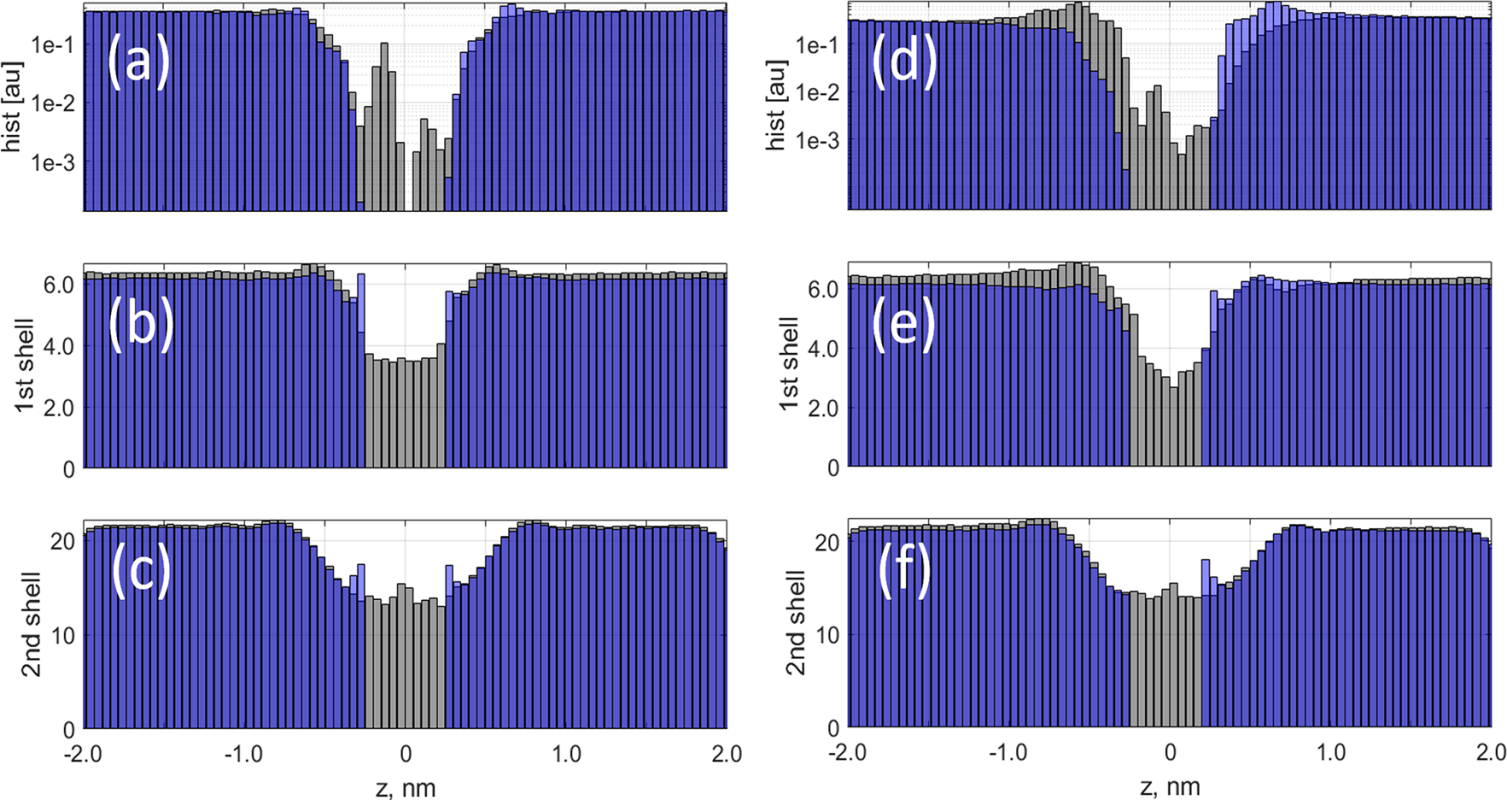
MD-simulated distributions of K^+^ (gray bars) and Cl^–^ (blue bars) ions in *z* direction (a, d); number of oxygen atoms in the first (b, e) and second (c, f) hydration shells of potassium (gray bars) and chloride (blue bars) or applied field (a) 25 and (b) 200 mV/nm.

In addition to the simple geometry-based estimates provided in subsection 4.3, in particular eq 5, the dehydration barrier can also be estimated by the use of MD simulations, which enable tracking of the time-dependent changes of the water environment of K^+^ ions as they traverse the pores. The results of such a calculation are shown in Figure 9 for two different values of the applied field. As shown in the figure, when an ion is approaching the pore, the 1st hydration shell can lose up to 50% of its water, while the 2nd shell can be reduced by up to 35%. We note that these values are consistent with earlier work describing similarly sized pores.^25^ The resultant dehydration barrier can then be estimated by using an equation similar to eq 5,
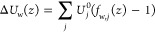
9where *f*_w,*j*_ is the fraction of the water molecules remaining in the shell as a function of distance from the membrane. Importantly, the results obtained demonstrate that the number of water molecules in the dehydrated shells depends on the applied field.

**Figure 10:**
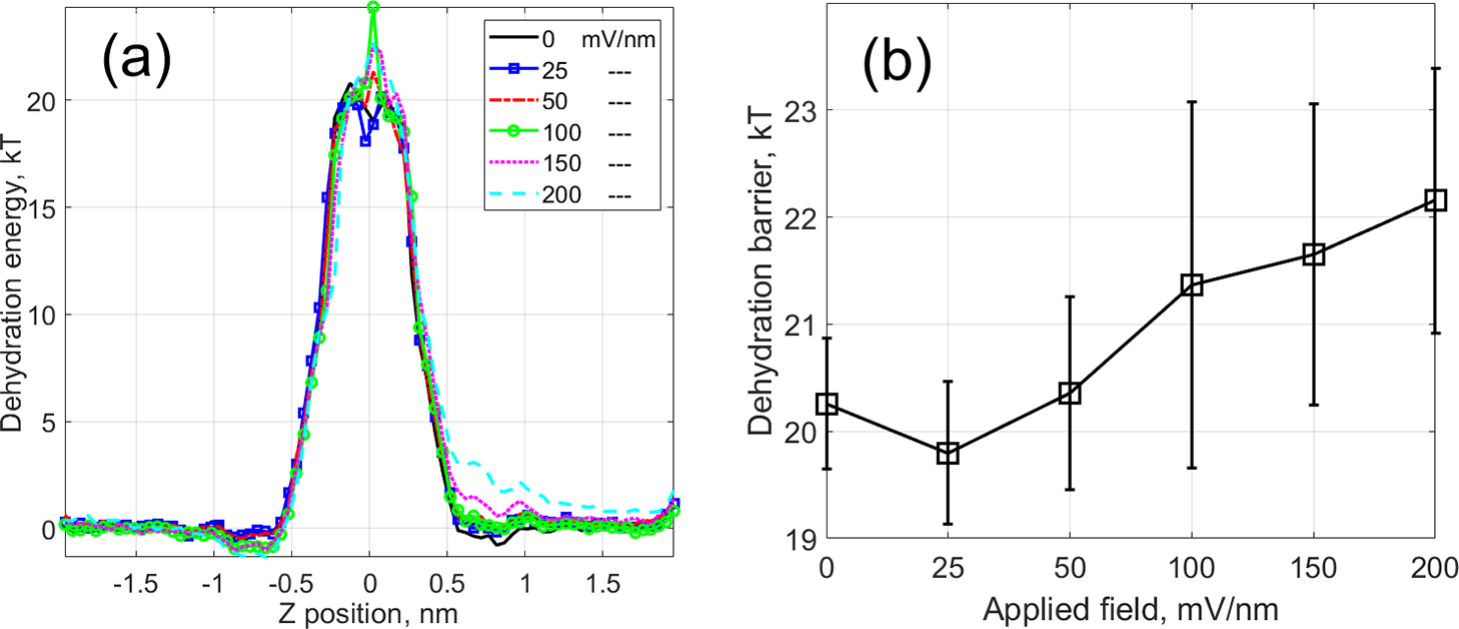
(a) Dehydration energy Δ*U*_w_(*z*) obtained in MD as a function of position *z* for different applied fields shown in the legend in mV/nm. (b) Field-dependence of the dehydration barrier. The squares show the mean value of the barrier for the *Z* position between −0.22 and 0.22 nm. The bars in (b) are the corresponding standard deviations.

The dehydration barrier is shown in Figure 5b by a dashed black line. The fluctuations at the top of the curve are once again attributable to the poor statistics of ions in the membrane plane. We can see that the estimate of the dehydration barrier based on shell geometry agrees with that based on the MD-simulated data for zero applied field. We note that the key advantage of an MD-based barrier estimation is that it can be used in the presence of an applied electric field. The results of such estimations are shown in Figure 10a. There are two notable features: the dehydration barrier in the presence of bias is slightly asymmetric with respect to the pore location, and this asymmetry increases as a function of increasing applied field.

The height of the dehydration barrier also increases with increasing applied field, as shown in Figure 10b. The large uncertainties correspond to the fluctuations of the barrier height observed between ±0.22 nm. Although not surprising, this observation serves as a reminder that the assumption of bias-independent barriers in simulations is generally incorrect and all comparisons between MD-simulated or experimental data and any perturbative analytical estimates should be made carefully, as the direct comparison is in principle only possible at zero or low biases. The observed dependence on the applied field of the number of water molecules in the dehydrated shells, as well as the corresponding increase in the dehydration barrier, is relatively weak. Here we call it the weak 1st mechanism of field-induced dehydration.

**Figure 11:**
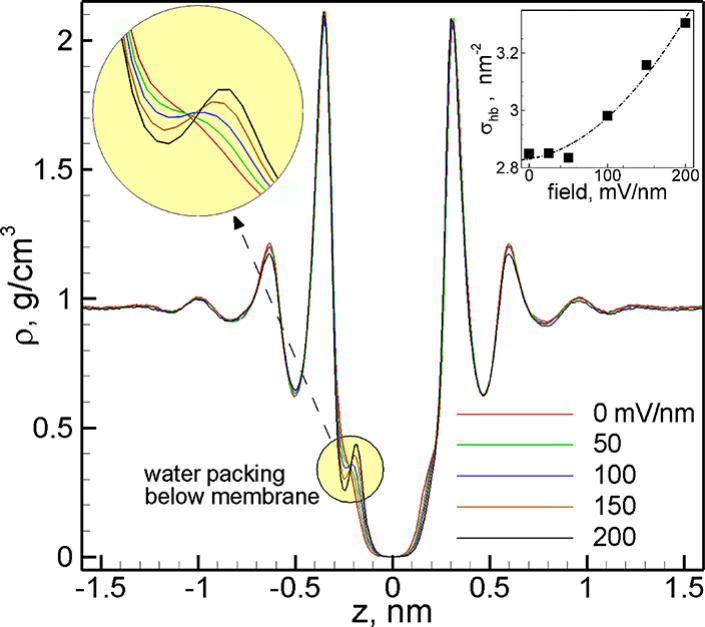
Time-averaged water density within *XY* slices as a function of the *Z* position of each slice for various values of applied field. The top-right inset shows the time-averaged sheet density of water–C_2_N hydrogen bonds (σ_hb_). As shown, the membrane is located at *Z* = 0 nm.

At the same time, the physical mechanisms leading to the field dependence deserve a more detailed discussion, as provided in the next section. We note that the distribution of water molecules in the hydration shells was assumed to depend only on the number of water molecules surrounding the ion at any given time,^4,24−26^ but this assumption is also incorrect because the shape of the hydration shell can contribute to barrier modification and is generally a function of the applied field. It is worth noting that, beyond their effect on the individual hydration shells, large applied fields are expected to modify the solvent–membrane interactions in general. For instance, as shown in Figure 11, the system-wide water distribution is mostly field-independent at the field magnitudes considered. However, field dependence is clearly evident at the membrane–water interface below the membrane (*Z* ≈ −0.25 nm; see also the top-left inset). This field-dependent water packing at one of the membrane–water interfaces is accompanied by field-dependent hydrogen-bond formation between the solvent and the membrane. As shown in the top-right inset of Figure 11, the corresponding sheet density of water–C_2_N hydrogen bonds σ_hb_ increases with increasing applied field. Overall, such field-dependent solvent ordering can further contribute to the field dependence of dehydration barriers. Although relatively weak from the energetic standpoint, the observed effect is another example of bias dependence beyond the assumption of infinitesimal perturbation.

In bulk solution, the hydration shells are usually symmetric, and the corresponding average force that they exert on the ions is zero, corresponding to bulk diffusion. Close to the pores, however, in the absence of any external bias, this symmetry is briefly broken during permeation. Near the pore, in the presence of an external field, the symmetry with respect to the membrane plane is broken and the barrier is determined, not only by the total number of water molecules in the hydration shell but also by their distribution around the ion on either side of the membrane. In the limiting case of a large externally applied field and an ion located in the pore plane, the orientations of water molecules on either side of the membrane are asymmetric. A possible contribution to the local asymmetry at high fields may also arise from the quasi-ballistic motion of ions through pores, i.e., a purely dynamical effect arising from the finitely compressible shell surrounding an ion moving along a nearly straight path with large acceleration. Regardless of any dynamical effects, it is clear that an asymmetric shell will exert a considerable permeation-opposing force on the ion. We now consider these field-induced asymmetries in greater detail, as well as their effect on the entire system.

### 5.3 Field-Induced Asymmetry of Hydration Shells

**Figure 12:**
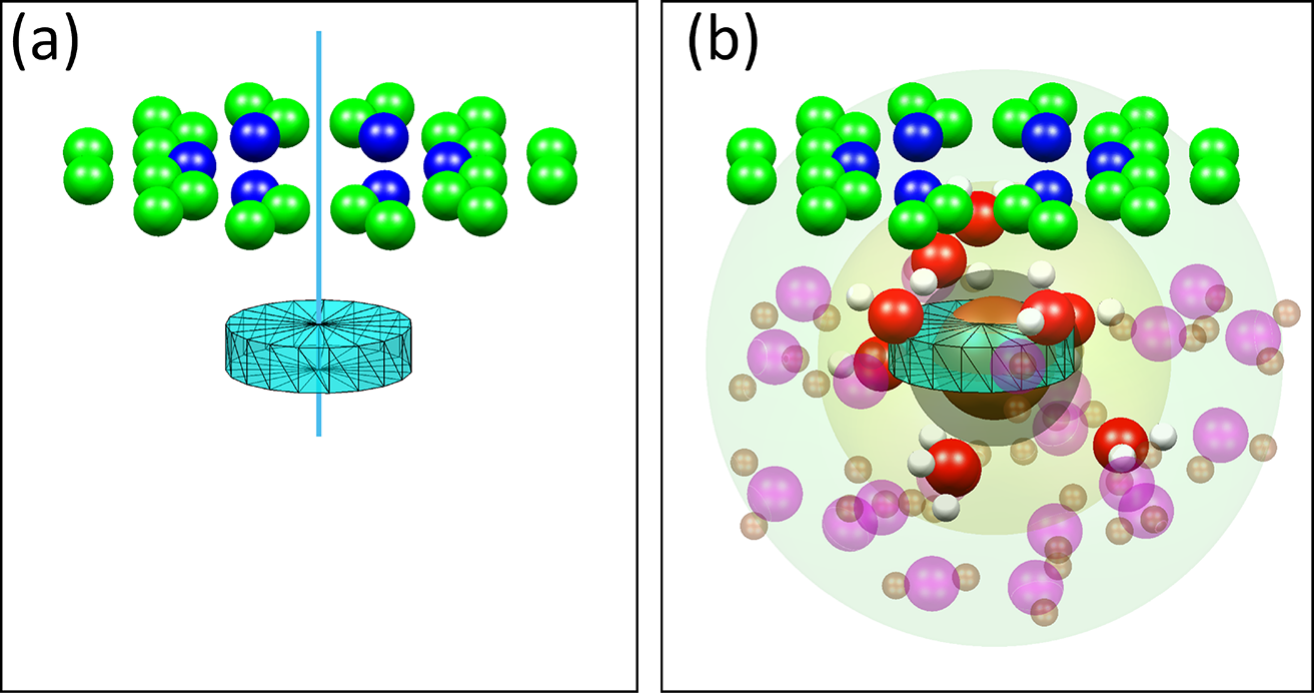
(a) Membrane, shown as a collection of carbon atoms (green) and nitrogen atoms (blue). The axis of the pore is shown by the vertical line. A disk-shaped bin located on the pore axis 0.45 nm below the membrane (height, 0.1 nm; radius, 0.2 nm) is shown by transparent faces shaded cyan. (b) Snapshot of hydration shells and water molecules around a K^+^ ion located inside the sampling bin. Color code: K^+^ ion (brown), oxygen atoms in the 1st shell (red), oxygen atoms in the 2nd shell (pink), hydrogen atoms in the 1st shell (white), and hydrogen atoms in the 2nd shell (gray). The transparent spheres show the effective boundaries of the hydration shells with radii <0.19 nm (gray), between 0.19 and 0.38 nm (yellow), and between 0.38 and 0.62 nm (green).

To analyze the asymmetry of the hydration shells, we consider distributions of oxygen and hydrogen atoms in the first two hydration shells of ions approaching and passing through the pore. To find these distributions, we use (cf. the calculations of the PMF in subsection 4.1) disk-shaped bins located on the pore axis at a series of *z* distances from the membrane; see Figure 12a. Next, we detect all ion–water configurations for the K^+^ ions located in each bin. Finally, for each ion’s position, we track coordinates of all the oxygen and hydrogen ions located within the 1st and 2nd hydration shells, as shown in Figure 12b. The number of such configurations varies from several thousand in the bulk to a few dozen near the pore.

Note that the effect of the system size can be obtained using radial distribution functions (RDFs)^8^ for symmetrical systems. However, to analyze the asymmetry of hydration shells, the RDF-based methods have to be substantially extended.^57^ Therefore, a conventional RDF-based analysis does not provide any additional information as compared with the MD-based approach adopted in this work.

**Figure 13:**
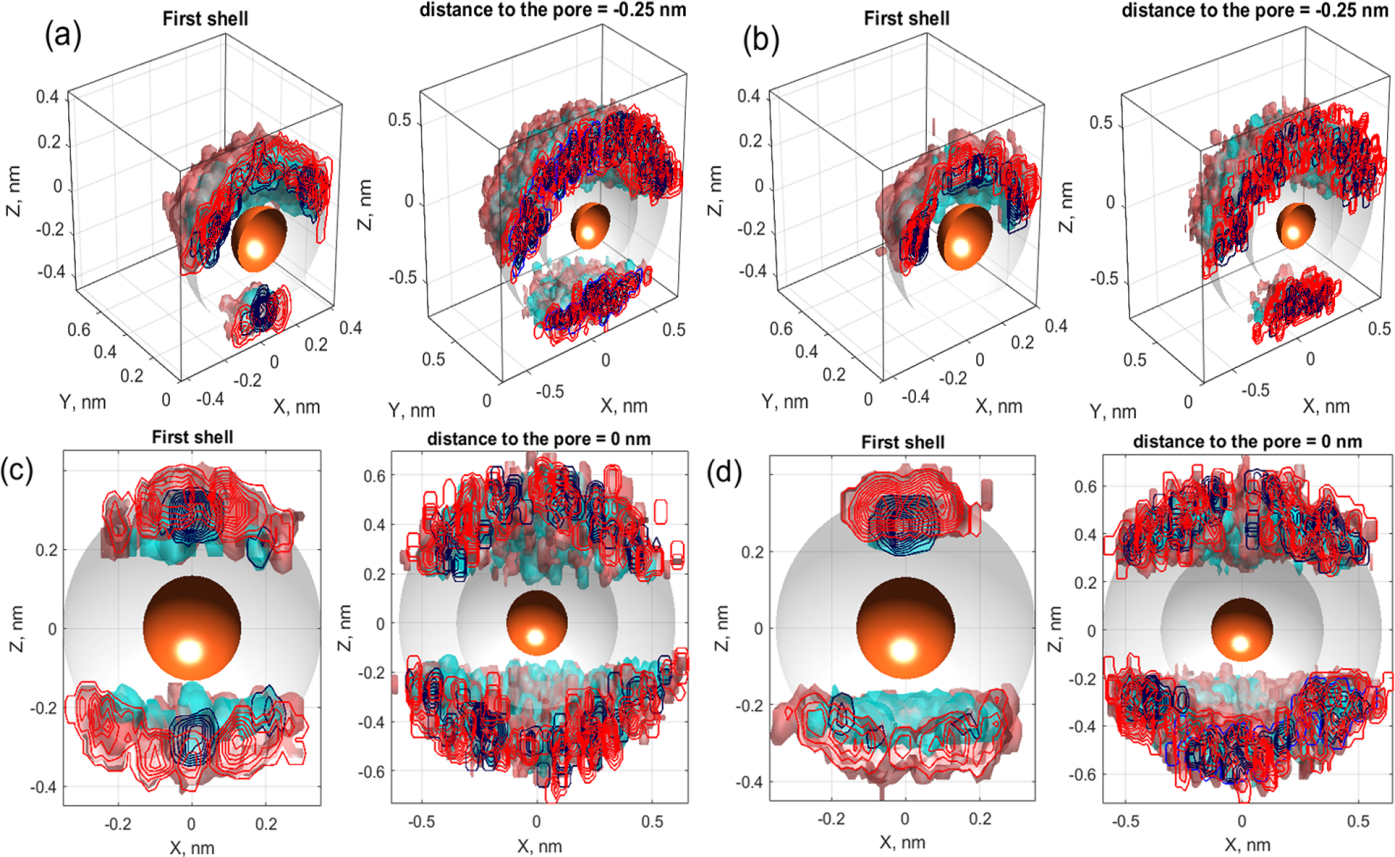
(Top tow) Distribution of the oxygen (blue) and hydrogen (red) atoms in the *XZ* plane for zero applied field (a) and 200 mV/nm (b). The distance of the ion from the membrane is 0.25 nm. (Bottom row) Distributions for an ion in the plane of the membrane for applied fields: (c) 0 V and (d) 400 mV/nm.

The radii of the shells are given in section 4.3. Each distribution was obtained from an 800-ns-long simulation, during which we averaged the coordinates of all oxygen and hydrogen atoms within the two first shells for an ion located in a cylinder of height 0.1 nm and radius 0.2 nm. Examples of such distributions for a cylinder located 0.25 nm above the pore are shown in Figure 13.

**Figure 14:**
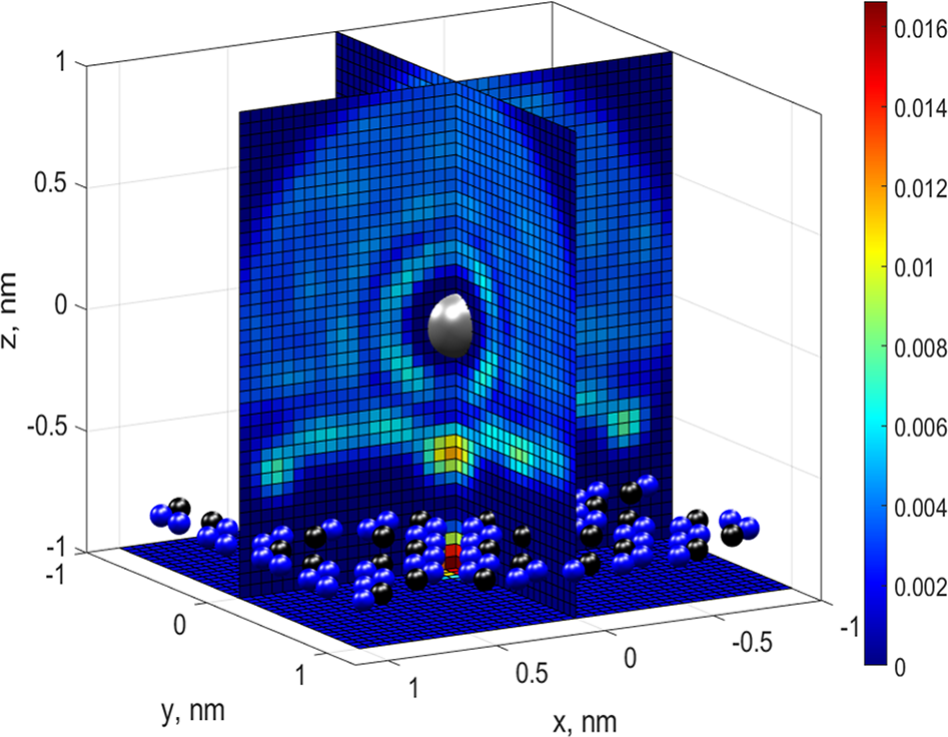
Oxygen atom distribution around a potassium ion shifted along the pore axis in the *z* direction by 0.75 nm. Carbon and nitrogen atoms in the C_2_N membrane are shown by blue and black colors, respectively. The potassium ion is shaded by a silver color.

In Figures 13 and 14, it can be observed that the hydration shells of ions passing through the pore at a nonzero applied field are generally asymmetric. This asymmetry depends on the distance to the pore and is an increasing function of the applied field. As mentioned earlier, the resulting asymmetric charge distribution, in addition to the dynamic effects, should induce transport-opposing Coulomb forces.

In particular, if we consider the distributions of oxygen and hydrogen atoms in the 1st and 2nd hydration shells in Figures 13b, we see that the water oxygens are located closer to the ion, predominantly in an asymmetric manner. Therefore, it is clear that this is a case where the averaged force will indeed oppose transport, readily contributing to the transition barriers and further increasing with increasing bias. For example, we note that, for *E*_app_ = 200 mV/nm, all oxygen atoms in the 1st hydration shell are located on one side of the ion. Similar changes can be observed for an ion located in the membrane plane, as shown from a different angle in Figure 13c and d. In this case, the distributions are symmetric for zero field and become strongly asymmetric under nonzero bias.

### 5.4 Field-Induced Forces Opposing Ion Transport

To estimate the electrostatic forces acting on the ion due to asymmetry of the hydration shells, and hence the resultant changes in the energy barriers, we find the charge distribution in the hydration shells as a function of the ion’s distance from the pore. To do so, we select a cube with 2.0 nm sides, centered at the ion location, and split the corresponding cubic volume using a 51 × 51 × 51 mesh. With the TIP4 model, oxygen atoms are each assigned a charge of −1.04*q*, while hydrogen charges are 0.52*q*. The total charge of each mesh volume is the sum of the probability of finding an oxygen atom and the probability of finding a hydrogen atom within this volume, weighted by the corresponding charges. For example, the probability of finding oxygen atoms is shown in Figure 14.

Here, the ion (silver sphere in the center of the cube) is located 0.75 nm above the pore. The pore is embedded into the C_2_N membrane with carbon and nitrogen atoms shown as blue and black spheres, respectively. It can be seen in the figure that the 1st hydration shell (light circle around the ion) remains almost intact. Although the 2nd shell is nearly intact in the upper hemisphere, it becomes fragmented in the vicinity of the pore below the ion.

**Figure 15:**
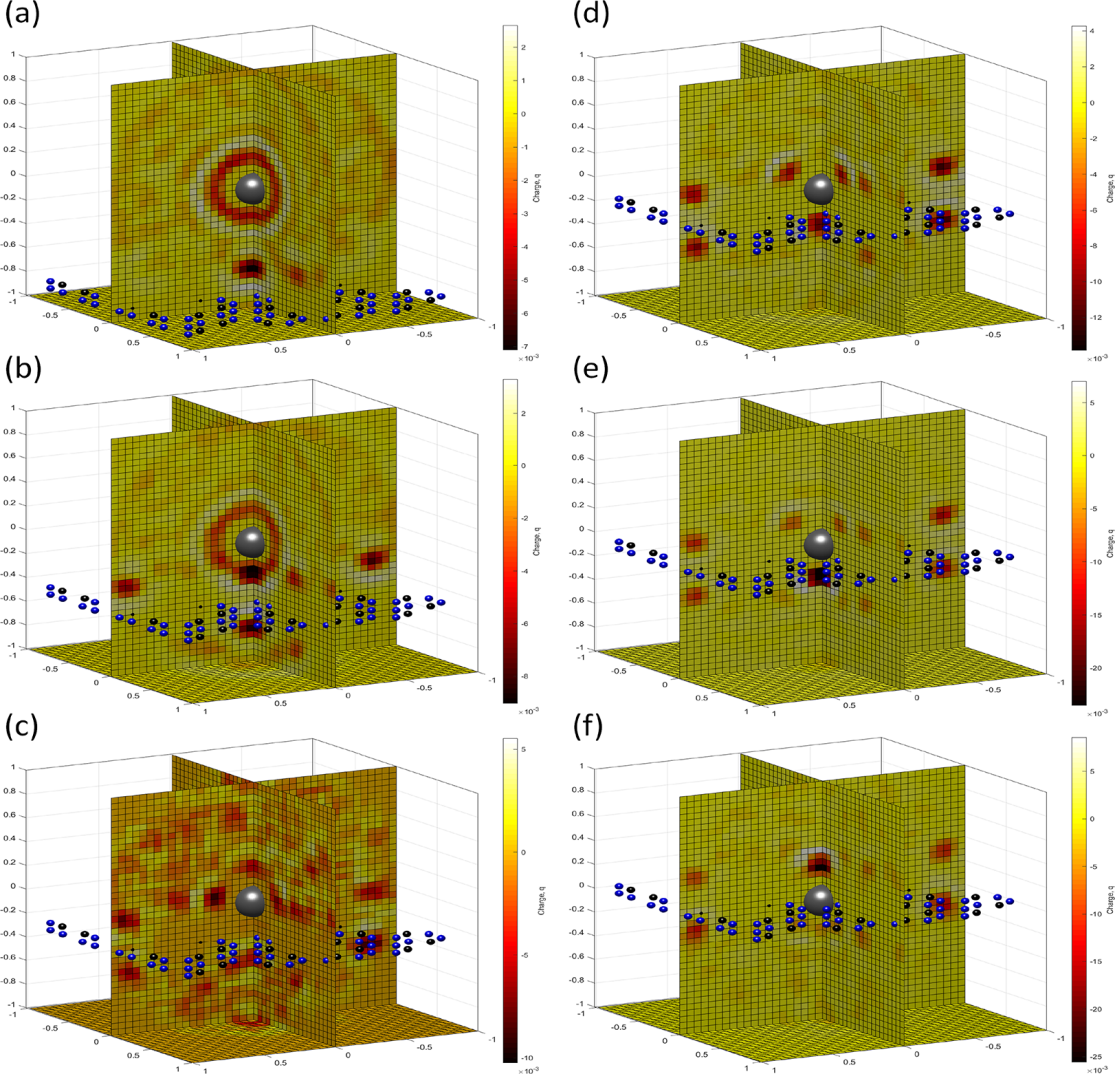
Charge distribution around a potassium ion at different *z* positions along the pore axis: (a) *z* = 0.9 nm; (b) 0.5 nm; (c) 0.3 nm; (d) 0.2 nm; (e) 0.1 nm; and (f) 0 nm. Carbon and nitrogen atoms in the C_2_N membrane are shown by the blue and black colors, respectively. The potassium ion is shaded by a silver color.

By superimposing this distribution upon the probability of finding hydrogen atoms weighted with 0.52*q*, we obtain the distribution of the total charge in two hydration shells of the ion. The evolution of these charge distributions as a function of the ion’s distance from the pore is shown in Figure 15. From Figure 15a we see that, at large distance from the pore, two nearly intact charged rings (negative and positive shown as red and white, respectively) surround the ion as expected.^24^ When the ion approaches the pore, the charge distribution around the ion becomes strongly asymmetric/fragmented, leading to a rapidly increasing field-induced electrostatic force. The maximum force corresponds to the maximum asymmetry and arises at positions about ±0.25 nm from the pore. The resultant Coulomb force was calculated as
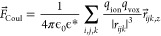
10where |*r*_*ijk*_| is the distance between the ion and the voxel with indices *i*, *j*, and *k*, the summation is over all voxels, and *q*_ion_ and *q*_vox_ are the ionic and voxel charges, respectively. Here, we are interested in the *z* component of the force. For consistency, the value of ϵ* in this equation is taken to be the same as ϵ_p_ = 3 in eq 4. We note once again that this is a crude approximation and that a more accurate calculation of the values of effective dielectric permittivity ϵ*(*r*) in confinement is required. Moreover, the effects of shell asymmetry may indeed introduce a second-order asymmetry in the distribution of the local dielectric permittivity itself, further contributing to the *z* component of the resulting force.

**Figure 16:**
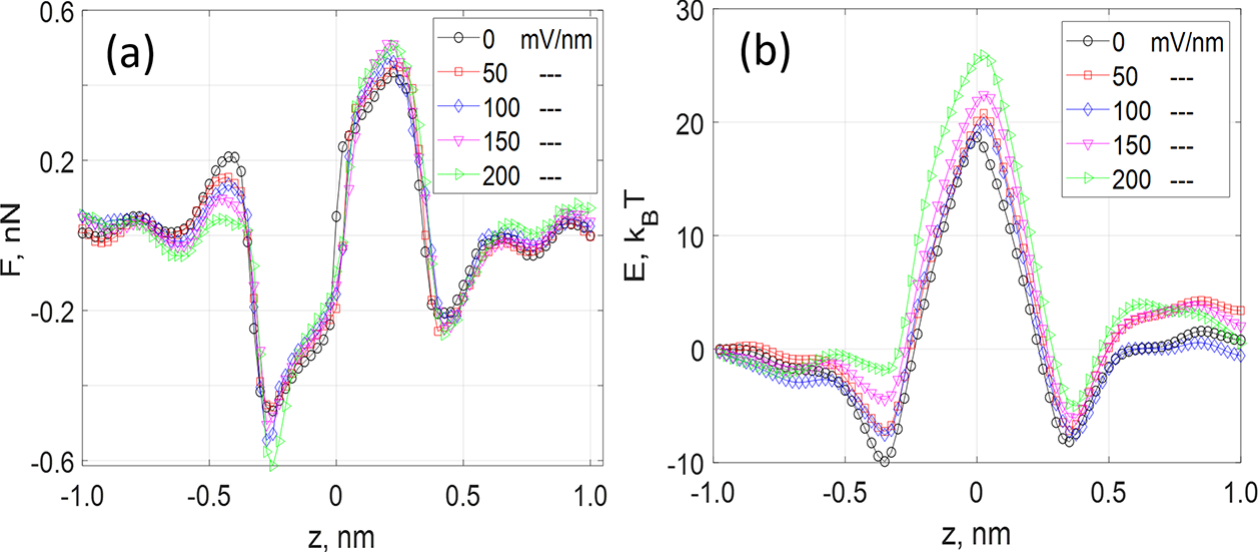
Coulomb force (a) and energy barrier (b) due to the charge distribution around a potassium ion obtained in MD for various magnitudes of applied fields shown in the legends in mV/nm.

The *z* component of the force as a function of applied field is shown in Figure 16a. As expected, the force decreases to near-zero at a considerable distance away from the pore due to the symmetry of nearly intact hydration shells. The maximum shift in the force due to external bias is estimated at ∼0.1 nN between the unbiased case and a field of ∼200 mV/nm. To estimate the corresponding changes in the energy barrier, we integrate the force (eq 10) along the *z* coordinate. The result, shown in Figure 16b, clearly demonstrates the field-induced increase of the central barrier in the amount of ∼7 kT, which is significantly larger than the increase shown in Figure 10. One can also see the emergence and deepening of a local minimum in the quasi-potential^58^ located at approximately −0.75 nm, which is also in agreement with the data shown for Δ*U*_w_(*z*) in Figure 10 (left).

We note that the asymmetry-induced barrier enables one to reveal an additional feature that is missing in the local minimum in Δ*U*_w_(*z*) . Namely, one can clearly see in Figure 16b that the local minimum located at approximately −0.3 nm becomes shallow and disappears when the applied field is large enough. The change in location of this local minimum from approximately −0.3 to −0.75 nm with increasing field is presumably what underlies the switch of the optimal path between the two routes observed in Figure 2. The field-induced dehydration discussed in this section is relatively strong (cf. 1st mechanism discussed in section 5.2), and we call it the 2nd mechanism of the field-induced dehydration.

### 5.5 Mechanisms of Field-Induced Modifications of Barriers

We have identified two mechanisms underlying the field-induced barrier that opposes the conduction of ions through the pore. The first mechanism discussed in section 5.2 is relatively weak and corresponds to the dependence of the ion’s dehydration in the pore on the applied field, i.e., because the number of water molecules in the first shell is reduced when the field is increased. For example, the value of the barrier is increased by ∼2*kT* for an applied field of 200 mV/nm. The second mechanism described in this section is considerably stronger and corresponds to the field-induced asymmetry of the oxygen distribution in the first two hydration shells. The corresponding barrier increase is ∼7*kT* for an applied field of 200 mV/nm.

Note that we only considered here electrostatic interactions of the permeating ion with the hydration shell determined by eq 10, while asymmetry of the shell also affects the ion’s electrostatic interaction with charged atoms on the rim and the Lennard-Jones interaction with the shell and rim atoms. Importantly, it is the field-induced changes in the ion’s electrostatic interaction with the asymmetric shell that, together with the field-induced dehydration (see section 5.2), comprise the two mechanisms that oppose an increasing current through the pore with increasing applied field. In a sense the field-induced asymmetry and dehydration of the shells of the permeating ion represent the effect of dielectric medium polarization near the membrane.

We propose that the increased dehydration cost is associated with the field-induced asymmetry of the shells. Note that the force estimates shown in Figure 16a are close to our earlier estimates, 0.35 nN per particle, of the maximal total electrostatic force experienced by an ion near a graphene nanopore.^47^ It is also worth noting that the field-induced asymmetry of the hydration shells may be strongly correlated with the local polarization of water molecules under the incident field, as discussed earlier.^41^ The two mechanisms leading to field-induced barrier changes oppose the original bias field and consequently limit transport, providing yet another example of solvent screening at the nanoscale. Interestingly, field-induced asymmetry of hydration shells along the *z* direction may be combined with in-plane manipulation of the shells induced by directed membrane strains,^59^ potentially yielding a path toward hybrid mechano-electric gating.

The latter phenomena partially compensate for the decrease of the electrochemical barrier, as discussed in subsection 4.4. We therefore attribute the deviation of the *I*–*V* curve predicted by eq 8 from the MD simulation to the field-induced asymmetries and field-induced changes in the dehydration barriers. We note that the changes in the hydration shells to oppose the effect of the applied field on the ion transport are consistent with the general statement of Le Chatelier’s Principle. In our case, if a dynamic equilibrium is disturbed by an external bias, the position of the new quasi-equilibrium is such that the effect of the bias is reduced.

The asymmetry of the shells also reveals the relationship between the ion’s dehydration and the electrostatic interaction with the charged pore. Such an interrelation, often neglected within the conventional approach (see ref (24) and eq 4 in section 4), comes into play due to the asymmetry-induced strong dependence of the local effective dielectric permittivity ϵ* on the ion’s distance from the membrane.^19,60^ This dependence (also evident from the estimates of ϵ* shown in the inset of Figure 6) strongly affects the local electrostatic interaction of the ion with the pore. In turn, the latter interaction modifies the orientations^41^ and distribution of the water molecules in the shells. We emphasize that induced asymmetry of the hydration shells appears to be a generic feature of nanopores whose dimensions are comparable with the diameter of the hydrated ion: understanding and tuning these effects paves the way to controlling permeation in such nanopores.

## 6 Summary and Conclusions

It is common for particle-based simulations and experiments to be compared with analytic theories that are only applicable close to equilibrium or that assume material continuity in the vicinity of the pore. Strictly speaking, such comparisons are valid only for systems involving wide pores and subject to infinitely small bias, electrical or otherwise. This routinely results in difficulties in the interpretation of the simulated and experimental data. The nature of systems under nonzero and large applied biases is far from being well-understood. In this Article we have investigated the specific effects of applied fields on ionic transport through subnanometer pores. The results obtained are not specific to the ultrahigh-pore-density membrane (C_2_N) considered and are, in fact, applicable to subnanoscale pores in various 2D membranes quite generally, regardless of the pore spacing.

We identified field-induced changes in the ions’ escape paths and demonstrated that these changes are closely associated with field-induced shifting of transport barriers in the vicinity of the pores. To further analyze these effects, we considered the distributions of ions and water molecules near the pores and the structure of the hydration shells around the ions passing through the pore as a function of applied field. We showed that both the electric double layers and the water layers became strongly asymmetric in response to the external field. In particular, the individual hydration shells of the ions exhibited a similar asymmetry.

We demonstrated two transport-opposing mechanisms arising from bias-induced changes in the hydration shells. The first mechanism was relatively weak and corresponded to an overall decrease of water numbers caused by the field. The second mechanism was considerably stronger and corresponded to field-induced asymmetry in the ions’ first two hydration shells. The corresponding changes in the permeation barrier were estimated and shown to partially compensate for the effect of the applied field, essentially presenting yet another manifestation of local screening at the nanoscale. The latter effect was also observed in the MD-simulated current–voltage curves compared to numerical solutions of the corresponding 1D Nernst–Plank (NP) equation. It was shown that the NP model agreed well with *I*–V curves obtained in the MD simulations for lower biases. At larger fields, the discrepancies between the simulated currents and currents from the NP-based model increased as expected, highlighting the need for robust field-dependent corrections to the corresponding energy barriers.
